# Smoking-Stratified Signal Decomposition and Feature Selection for Never-Smoker Cancer Classification in a Combined Lung–Breast Metabolomics Cohort

**DOI:** 10.3390/ijms27167350

**Published:** 2026-08-17

**Authors:** Bharadwaj Popuri, Jean-François Haince, Rashid A. Bux, Guoyu Huang, Paramjit S. Tappia, Bram Ramjiawan, Maria Vaida

**Affiliations:** 1 Department of Data Science, Harrisburg University of Science and Technology, Harrisburg, PA 17101, USA; bpopuri@my.harrisburgu.edu; 2 BioMark Diagnostic Solutions Inc., Quebec, QC G1P4P5, Canada; jhaince@biomarkdiagnostics.com (J.-F.H.); ghuang@biomarkdiagnostics.com (G.H.); 3 BioMark Diagnostics Inc., Richmond, BC V6X2W2, Canada; rahmed@biomarkdiagnostics.com; 4 Department of Food & Human Nutritional Sciences, Faculty of Agricultural & Food Sciences, University of Manitoba, Winnipeg, MB R3E 0T6, Canada; ptappia@sbrc.ca (P.S.T.); bramjiawan@sbrc.ca (B.R.); 5 Department of Pharmacology & Therapeutics, Max Rady College of Medicine, University of Manitoba, Winnipeg, MB R3E 0T6, Canada

**Keywords:** smoking confounding, lysophosphatidylcholine, XGBoost, never-smokers, biomarker panel

## Abstract

Metabolomic cancer classifiers trained on mixed-smoking cohorts may embed tobacco exposure signal within their predictions, degrading performance in never-smokers, a population in which lung adenocarcinoma is frequently diagnosed. We developed a two-stage framework that (i) decomposes a shared 129-metabolite panel from a combined lung–breast cancer cohort (n=1038) into cancer-specific (Signal C), smoking-specific (Signal S), and shared (Signal S∩C) components using two-way analysis of variance with Benjamini–Hochberg correction, and (ii) applies multiple feature-selection strategies to identify the minimal Signal C subset that surpasses the all-metabolite baseline for never-smoker cancer detection. Two-way ANOVA partitioned 54 of 129 metabolites as cancer-specific (Signal C) and 57 as smoking-specific (Signal S), suggesting that nearly half of the shared panel is influenced by tobacco exposure. A model of 19 Signal C metabolites, selected by composite rank aggregation across four feature-selection methods and trained with gradient-boosted trees, achieved a never-smoker area under the receiver operating characteristic curve (AUC) of 0.907 on pooled out-of-fold predictions (0.910 as a mean across folds) against an all-metabolite baseline of 0.895, using 85% fewer metabolite measurements. The signal decomposition is a reproducible and interpretable way to identify metabolites whose case–control differences are not attributable to tobacco exposure, and it permits a substantial reduction in panel size.

## 1. Introduction

The utility of published biomarker panels is limited by a methodological blind spot. The majority of discovery cohorts contain a mixture of smokers and never-smokers, and tobacco smoke induces perturbations across lipid, amino acid, and organic acid metabolism that overlap substantially with cancer-associated signatures [1,2]. When a classifier learns from a mixed cohort, it encodes a composite of cancer signal and smoking signal. Applied to a never-smoker, the smoking component is absent, reducing sensitivity in a population that nonetheless bears meaningful cancer risk.

This problem is particularly acute for lung cancer in never-smokers, which accounts for approximately 15–20% of all lung cancer cases and represents a biologically distinct entity characterized by a higher prevalence of EGFR mutations, female sex, and East Asian ethnicity [3,4]. Never-smoking Asian women with lung adenocarcinoma are thus precisely the population most likely to receive a false-negative result from a classifier trained on tobacco-exposed cohorts. Despite a weaker and more contested causal relationship with smoking, breast cancer metabolomics studies frequently include smokers and non–smokers without stratification, and several lipid biomarkers documented in breast cancer literature, including lysophosphatidylcholines (LysoPCs) and sphingomyelins, show significant concentration differences attributable to smoking status [5,6].

Lung and breast carcinomas are biologically distinct diseases with different tissue origins, driver mutations, and metabolic programs. A single plasma metabolite panel is not expected to capture tumor-type-specific biology equally well in both. The combined design was adopted for three methodological reasons.

First, the research question concerns the *separability of tobacco exposure from malignancy* in plasma metabolomics, not the discrimination of one tumor type from another. Answering it requires a cohort in which cancer status and smoking status vary independently enough to be identifiable in a factorial model. Neither cohort provides this alone. The lung cohort is 82.5% ever-smokers and the breast cohort 34.9%, so within either cohort the cancer and smoking factors are strongly collinear. Pooling them breaks that collinearity and yields a design in which all four cancer × smoking cells are populated, which is a precondition for the two-way ANOVA decomposition that follows. Second, both cohorts were profiled on the same targeted platform (Biocrates AbsoluteIDQ p180) in the same laboratory, so the 129 shared metabolites are measured on a common analytical basis. Third, the analysis is explicitly aimed at the metabolic signal that is *shared* across tumor types. Metabolites that discriminate cases from controls in both a lung and a breast cohort are, by construction, less likely to encode tissue-of-origin biology and more likely to reflect generic malignancy-associated processes such as altered fatty-acid oxidation, one-carbon flux, and phospholipid turnover. A panel restricted to such metabolites is the appropriate substrate for a smoking-independence analysis.

**Figure 1:**
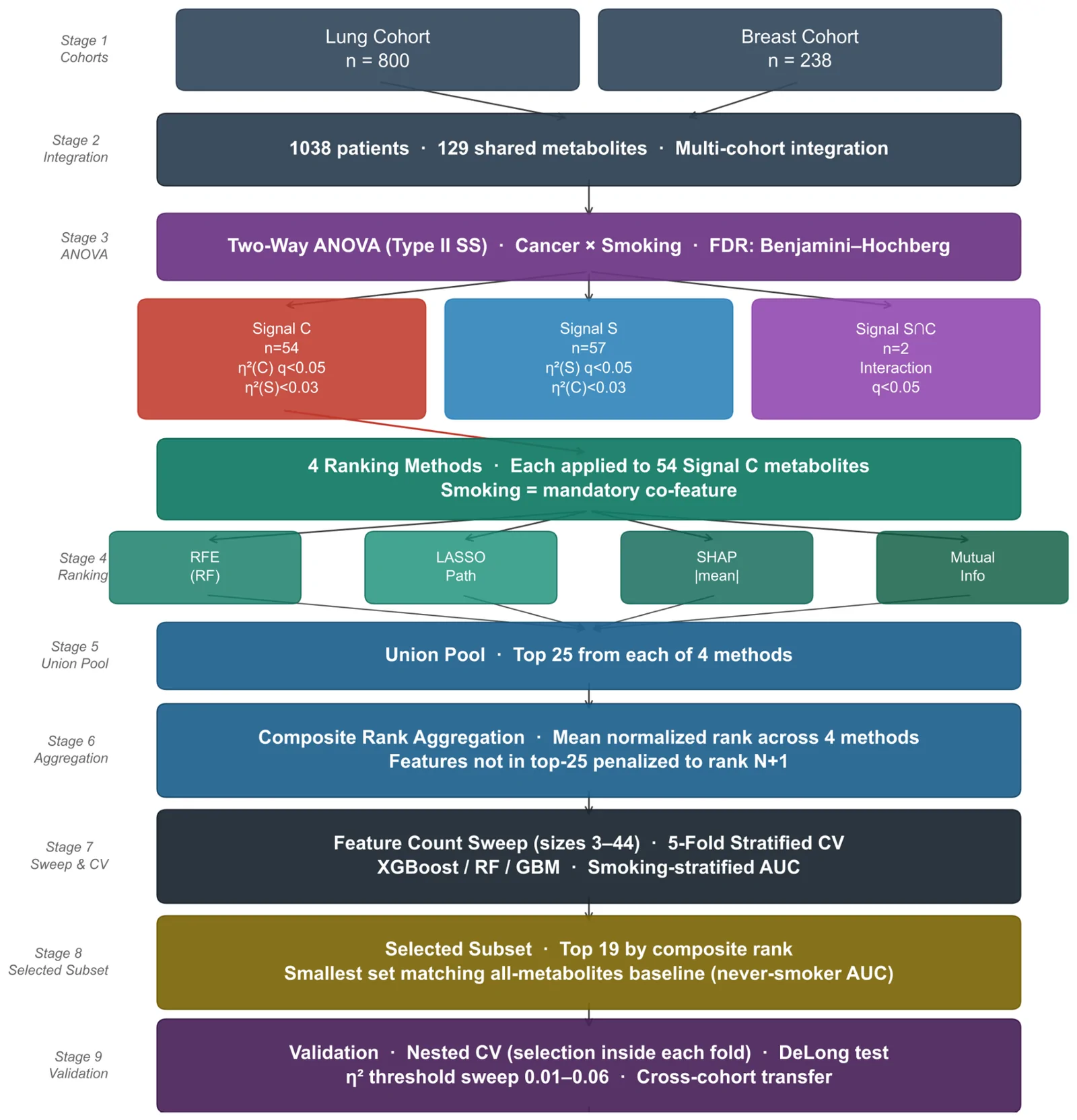
Full analytical pipeline used to identify cancer-specific metabolite signals independent of smoking effects.

We address this gap through a two-stage analytical framework applied to a combined 1038-patient cohort incorporating lung and breast cancer cases with complete smoking history (Figure 1). First, we decompose the shared 129-metabolite panel using two-way ANOVA, partitioning variance into cancer-attributable, smoking-attributable, and interaction components for each metabolite. Second, we apply four feature selection strategies, namely recursive feature elimination with cross-validation (RFECV), LASSO regularization path analysis, SHAP-based importance, and mutual information filtering, to identify a minimal Signal C subset and compare its never-smoker classification performance against that of the full unselected panel. Smoking status is retained as an input feature so that the classifier can use it as contextual information while relying on Signal C metabolites for cancer-specific discrimination.

## 2. Results

### 2.1. Dataset Characteristics

The combined lung–breast cohort comprised 1,038 patients, including 771 cancer cases (586 lung, 185 breast) and 267 controls. The 30% missingness filter was applied within each cohort before integration. The 129 metabolites that passed in both cohorts were carried forward and used in every model reported here. The smoking prevalence was markedly higher in the lung cohort (82.5%) than the breast cohort (34.9%), reflecting the epidemiological relationship between tobacco exposure and lung cancer. The combined dataset retained 743 ever-smokers (71.6%) and 295 never-smokers (28.4%), providing sufficient within-stratum sample sizes for stratified evaluation (Table 1).

### 2.2. Signal Decomposition by Two-Way ANOVA

**Figure 2:**
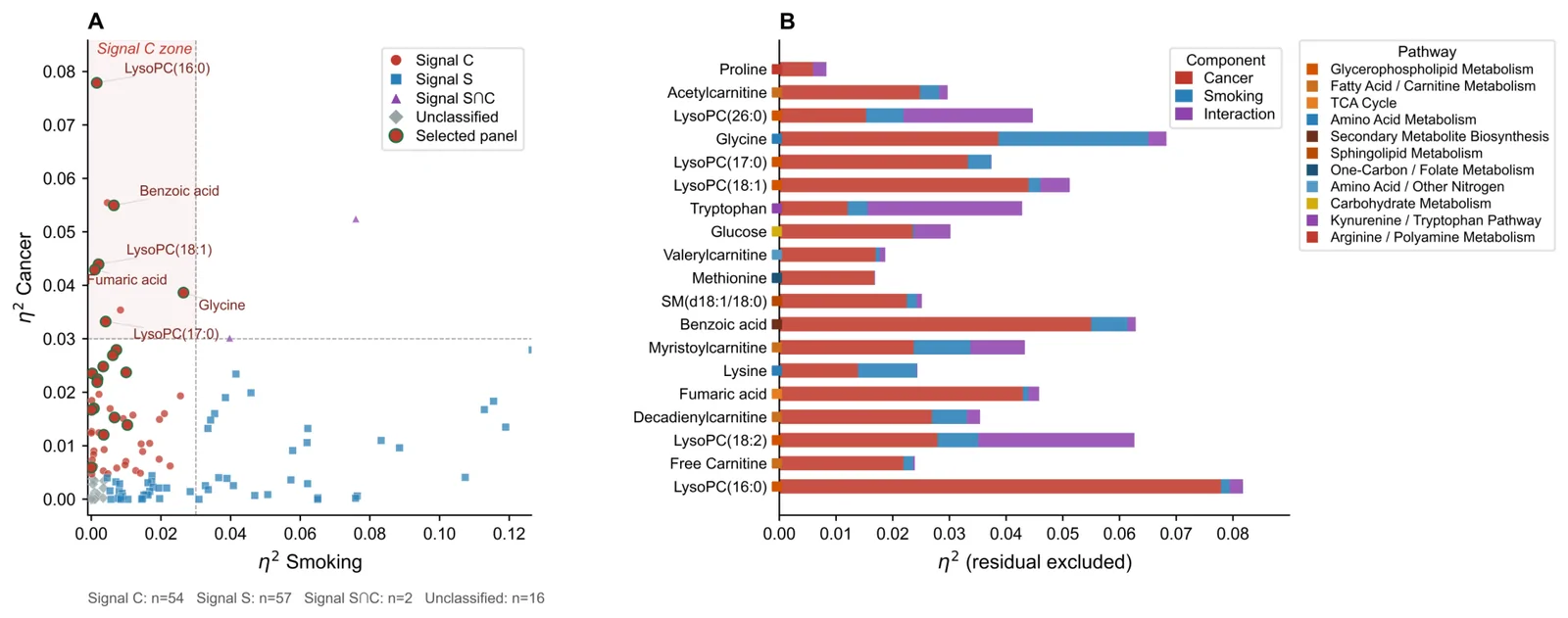
ANOVA signal classification and effect decomposition. (**A**) Scatter plot of metabolite effect sizes showing $\eta_{\mathrm{Cancer}}^{2}$ versus $\eta_{\mathrm{Smoking}}^{2}$. The decision boundary ($\eta^{2}$ threshold = 0.03) separates cancer-dominant (Signal C) from smoking-dominant (Signal S) features. Filled circles indicate the 19 selected panel metabolites. Signal C metabolites cluster at low smoking effect sizes and elevated cancer contributions, indicating smoking-independent cancer signals. (**B**) $\eta^{2}$ decomposition for the 19 selected panel metabolites in composite rank order shows that cancer effects dominate across features, with smaller smoking contributions and modest interaction terms.

Two-way ANOVA partitioned the 129 shared metabolites into four signal classes (Figure 2A): 54 metabolites were assigned to Signal C (cancer-specific, $q_{\mathrm{cancer}}<0.05$, $\eta_{\mathrm{smoking}}^{2}<0.03$), 57 to Signal S (smoking-specific), 2 to Signal S∩C (interaction-driven), and 16 remained unclassified. The near-equal split between Signal C and Signal S (41.9% vs. 44.2%) indicates that nearly half the shared metabolite panel carries smoking-attributable variance that may confound cancer classification in mixed-exposure cohorts.

The top-ranked Signal C metabolites by $\eta_{\mathrm{cancer}}^{2}$ were LysoPC a C16:0 ($\eta^{2}=0.078$, q<0.0001), Homovanillic Acid ($\eta^{2}=0.055$), Benzoic Acid ($\eta^{2}=0.055$), LysoPC a C18:1 ($\eta^{2}=0.044$), and Fumaric Scid ($\eta^{2}=0.043$). Smoking-attributable variance among these was low but not uniformly negligible ($\eta_{\mathrm{smoking}}^{2}=0.0010$ for Fumaric Acid, 0.0015 for LysoPC a C16:0, 0.0021 for LysoPC a C18:1, and 0.0065 for Benzoic Acid), in each case at least an order of magnitude below the corresponding cancer variance.

The all-metabolites plus smoking XGBoost baseline achieved an overall AUC of 0.922, a smoker AUC of 0.917, and a never-smoker AUC of 0.895. The AUC gap of +0.022 indicates that the baseline model generalizes less well to never-smokers than to smokers, a finding consistent with the hypothesis that the model encodes smoking-correlated variance that is absent in never-smoking test patients.

### 2.3. Sensitivity of the Signal Classification to the $\eta^{2}$ Threshold

The $\eta^{2}=0.03$ boundary separating Signal C from Signal S is a convention and not a value derived from theory (Section 4.2). Its effect on the results was assessed empirically at six thresholds spanning $\eta^{2}\in \left\{0.01,0.02,0.03,0.04,0.05,0.06\right\}$, with all other parameters held fixed (Table 2).

The size of the Signal C set depends moderately on the threshold. It ranges from 41 metabolites at $\eta^{2}<0.01$ to 63 at $\eta^{2}<0.06$, against 54 at the primary value. Panel composition is more stable than that range implies. Between 14 and 16 of the 19 primary panel metabolites are recovered at each threshold, and 12 are recovered at every threshold. These 12 are Benzoic Acid, C0, C2, C5, C10:2, C14, Fumaric Acid, Glucose, LysoPC a C16:0, LysoPC a C18:2, Methionine, and Proline.

Classification performance is stable across the range tested. Never-smoker AUC varied between 0.900 and 0.908, a spread of 0.009, and exceeded the all-metabolite baseline of 0.895 at all six thresholds. Overall AUC varied between 0.916 and 0.918. The primary threshold of 0.03 gave a never-smoker AUC of 0.907, which is neither the best nor the worst of the six. The AUC gap was negative at five of the six thresholds.

The behavior of the framework therefore does not depend on the particular cutoff chosen within this range. Re-ranking the Signal C set by $\eta_{\mathrm{cancer}}^{2}$ alone did not exceed the baseline at any threshold. The reported performance is a property of the full pipeline, specifically of the multi-method composite ranking.

### 2.4. Feature Selection Results

RFECV selected 30 features in total, comprising 29 Signal C metabolites plus the forced smoking covariate. Cross-validated AUC increased steeply from 3 to approximately 15 features, then plateaued, with diminishing gains beyond 20 features. The RFECV set included LysoPC a C16:0, Homovanillic Acid, Benzoic Acid, Fumaric Acid, Glycine, and multiple acylcarnitine species (C0, C2, C5, C14).

At the stability threshold of C=0.05, 22 Signal C features retained non-zero coefficients in the logistic regression selector. LysoPC a C16:0 exhibited the largest coefficient magnitude (β=−0.673 at C=0.05), with a negative sign indicating lower concentrations in cancer cases, consistent with LysoPC literature in lung adenocarcinoma [7,8,9].

Thirty-three Signal C metabolites satisfied the criterion MI(cancer)>MI(smoking) in the MI selector. LysoPC a C16:0 exhibited a high MI ratio of $5.2\times 10^{7}$, indicating essentially zero mutual information with smoking and maximal cancer-specific information content. Methionine (ratio = 7.6) and LysoPC a C18:2 (ratio = 27.7) also had MI ratios above unity.

**Figure 3:**
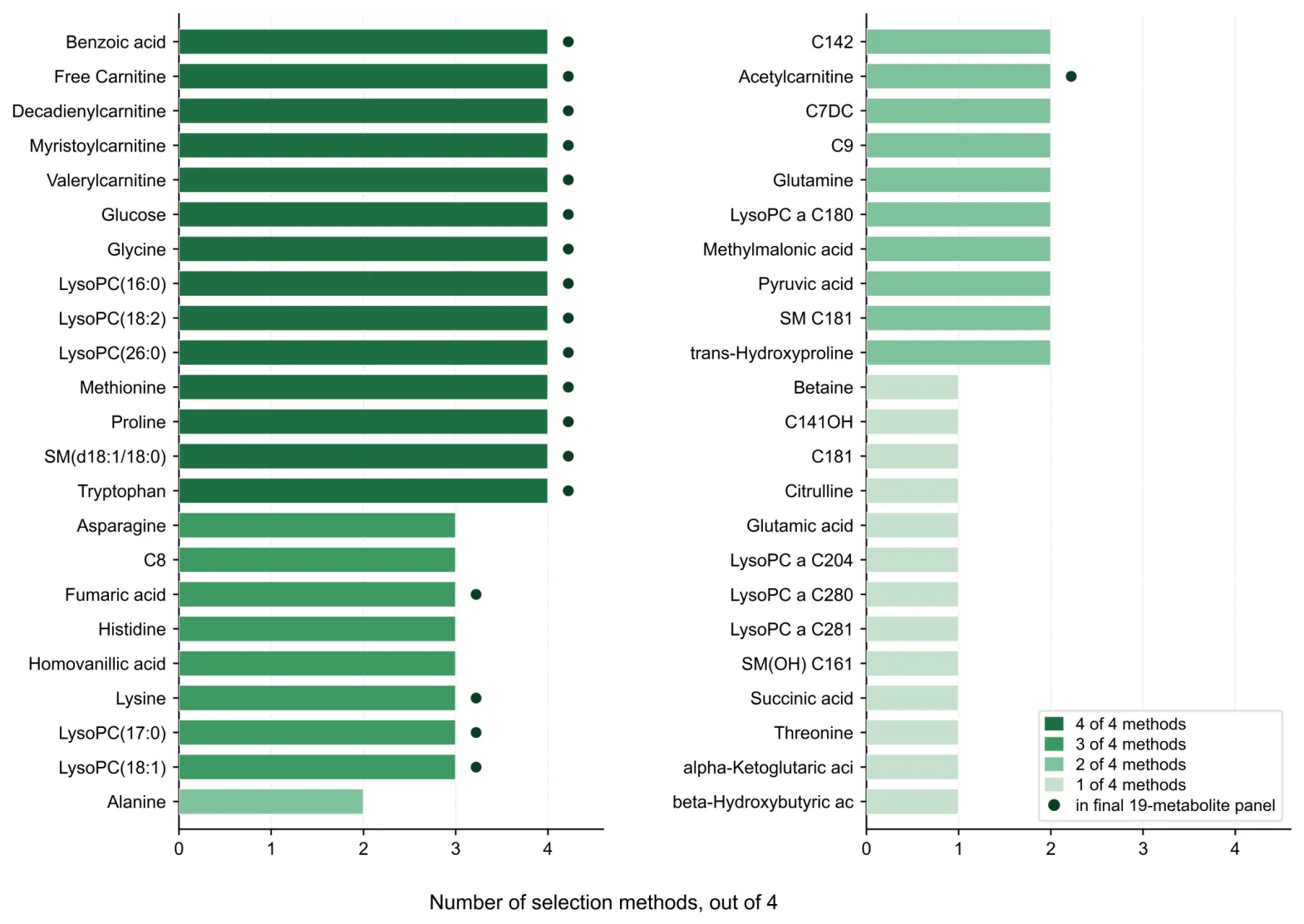
Consensus feature selection across four methods. Horizontal bars show the number of methods (RFECV, LASSO path, SHAP, mutual information) selecting each Signal C metabolite.

Fourteen metabolites were selected by all four methods: C0 (carnitine), C5 (valerylcarnitine), Glycine, Benzoic Acid, LysoPC a C16:0, Methionine, SM C18:0 (Sphingomyelin), Tryptophan, LysoPC a C26:0, C14 (Myristoylcarnitine), LysoPC a C18:2, Glucose, Proline, and C10:2 (Figure 3). The ≥3/4 consensus panel contained 22 metabolites.

**Figure 4:**
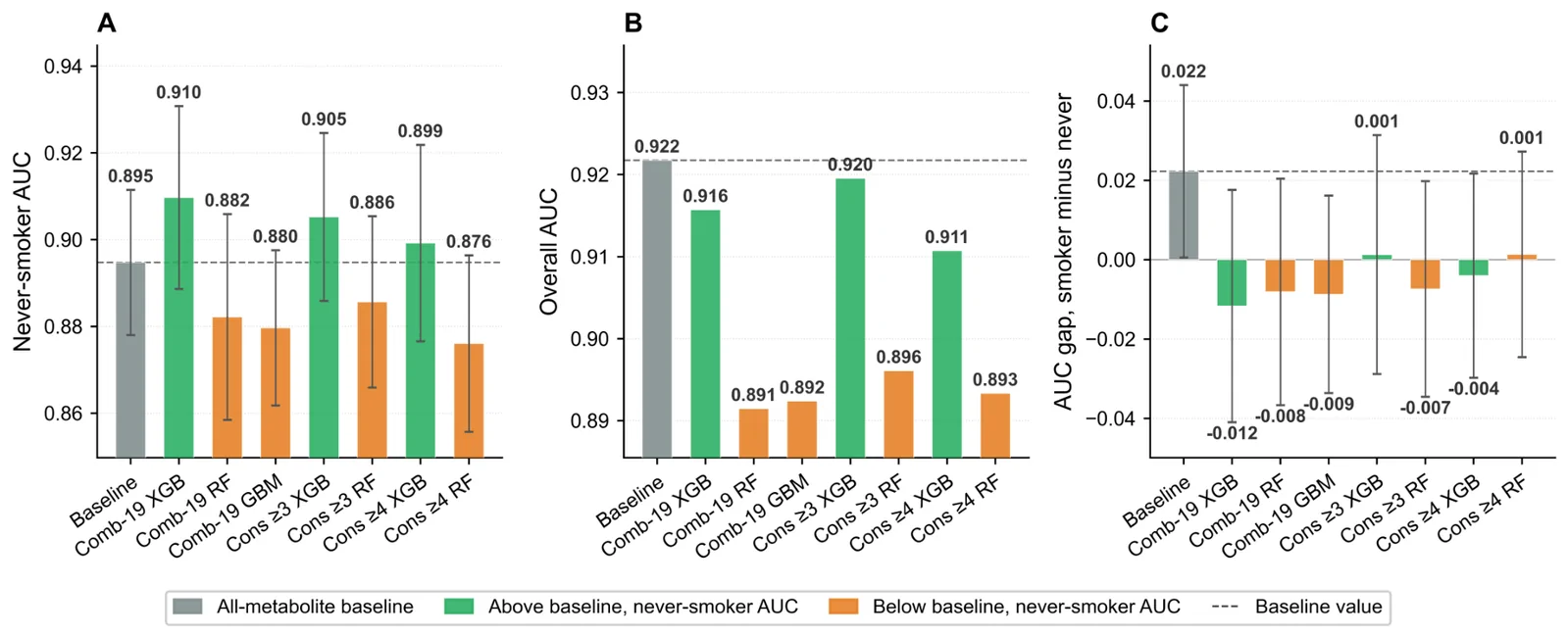
Composite-rank selected Signal C subsets versus all-metabolite baseline. Three-panel comparison of (**A**) never-smoker AUC, (**B**) overall AUC, and (**C**) AUC gap across evaluated model configurations. Green bars indicate never-smoker AUC exceeding the baseline dashed line. Orange bars indicate below baseline. The optimal composite-rank XGBoost model (composite top-19, 19 features + smoking) achieves never-smoker AUC = 0.910 (**A**, leftmost green bar) and an inverted AUC gap of −0.012 (**C**). Error bars in (**A**,**C**) show standard error across five cross-validation folds and overlap the baseline in every case.

Composite rank aggregation of the four method-specific rankings yielded a candidate pool of 44 unique metabolites (union of the top-25 from each method). Feature-count sweep evaluation on this combined ranked list identified multiple configurations exceeding the baseline never-smoker AUC of 0.895 (Figure 4).

Each of the four single-method ranked lists also produced at least one configuration exceeding the baseline, at best feature counts of 30 (RFE, never-smoker AUC 0.913), 21 (SHAP, 0.915), 38 (MI, 0.906), and 44 (LASSO, 0.910). Two of these, SHAP at 21 features and RFE at 30, reached a marginally higher never-smoker AUC than the composite-rank panel described below. The composite panel was nonetheless carried forward as the primary configuration because it is the most parsimonious of the configurations that exceed the baseline and because its membership is supported by more than one selection method, and the differences between these configurations are within the range attributable to fold assignment.

The configuration carried forward comprised 19 Signal C metabolites selected by composite rank aggregation plus smoking status, evaluated with XGBoost (Table 3). The selected metabolites included LysoPC a C16:0, Free Carnitine (C0), LysoPC a C18:2, Decadienylcarnitine (C10:2), Fumaric Acid, Lysine, Myristoylcarnitine (C14), Benzoic Acid, SM C18:0 (Sphingomyelin), Methionine, Valerylcarnitine (C5), Glucose, Tryptophan, LysoPC a C18:1, LysoPC a C17:0, Glycine, LysoPC a C26:0, Acetylcarnitine (C2), and Proline. The best subset used 85.3% fewer metabolite features than the baseline (19 vs. 129). The composite-ranked XGBoost configuration at n=19 Signal C metabolites achieved never-smoker AUC = 0.907, exceeding the baseline by 0.012 AUC points. The AUC gap inverted to −0.012 (Table 3).

**Table 3:** Performance comparison of baseline and feature-selected models on the combined multi-cancer cohort (n=1038, five-fold stratified cross-validation). All feature sets included the smoking variable. The AUC gap is defined as smoker AUC minus never-smoker AUC. Negative values indicate better performance in never-smokers. Values are means across the five folds.

Model	Features + Smoking	Overall AUC	Smoker AUC	Never- Smoker AUC	AUC Gap
All metabolites, XGB	130	0.922	0.917	0.895	+0.022
Top-19, XGB	20	0.916	0.898	0.910	−0.012
Top-19, RF	20	0.891	0.874	0.882	−0.008
Top-19, GB	20	0.892	0.871	0.880	−0.009
Consensus ≥3/4, XGB	23	0.920	0.906	0.905	+0.001
Consensus ≥3/4, RF	23	0.896	0.879	0.886	−0.007
Consensus ≥4/4, XG	15	0.911	0.895	0.899	−0.004
Consensus ≥4/4, RF	15	0.893	0.877	0.876	+0.001

Figure 4 presents the three-panel comparison of never-smoker AUC, overall AUC, and AUC gap. The composite-rank XGBoost model (19 features + smoking) exceeds the baseline on never-smoker AUC. Overall AUC declined relative to the baseline for every feature-selected configuration, the closest being the consensus ≥3/4 XGBoost model at 0.920 against 0.922, so the procedure did not improve cancer discrimination across the full cohort. It redistributed performance between the smoking strata. Standard errors across folds (Figure 4A,C) overlap the baseline for every configuration. The sign of the AUC gap in the consensus configurations was not stable across repeated executions and is therefore not interpreted here.

Because the never-smoker AUC difference between the baseline and the selected panel is small in absolute terms (0.895 vs. 0.910), we compared the two models using out-of-fold predicted probabilities for never-smokers pooled across the five folds. The two correlated ROC curves were compared using the DeLong test, with a 2000-replicate stratified bootstrap to obtain a confidence interval for the AUC difference. On pooled out-of-fold predictions the never-smoker AUC was 0.895 for the baseline and 0.907 for the selected panel, a difference of ΔAUC=0.012 (95% bootstrap CI −0.013 to +0.039; DeLong p=0.375). The confidence interval includes zero and the difference does not reach nominal significance. The point estimate is smaller than the 0.015 obtained by averaging fold-wise AUCs (Table 3).

The corresponding comparisons in the other strata run in the opposite direction. In ever-smokers the baseline achieved 0.917 against 0.897 for the selected panel, a difference of −0.020 (p=0.048 uncorrected), and across the full cohort the difference was −0.007 (p=0.328). Because three strata were tested, these *p*-values were corrected by the Benjamini–Hochberg procedure, giving q=0.375 (never-smokers), q=0.144 (ever-smokers), and q=0.375 (overall).

No stratum shows a statistically significant difference between the two models after correction. The selected 19-metabolite panel is therefore not distinguishable from the 129-metabolite baseline in any subgroup, in either direction, at the sample sizes available. The inversion of the AUC gap in Table 3 reflects a combination of a small non-significant gain in never-smokers and a somewhat larger non-significant loss in ever-smokers, and should not be interpreted as evidence that Signal C restriction improves never-smoker discrimination specifically.

### 2.5. Cross-Validated Feature Selection

In the primary analysis the signal decomposition and the four selection procedures were applied to the complete dataset before cross-validation, which is known to bias performance estimates upward. To quantify that bias, the decomposition, the four selection methods, and the composite rank aggregation were repeated independently within each training fold of an outer five-fold stratified cross-validation. The panel size of 19 was held fixed, so the nesting covers which metabolites are chosen and not how many.

Under the nested protocol the selected-panel never-smoker AUC was 0.901 (SE 0.019 across folds) against 0.910 under the non-nested protocol, an optimism of 0.009 AUC points. The nested baseline was 0.895 (SE 0.017), unchanged because the baseline involves no selection step. The optimism is therefore small, and the non-nested estimates in Table 3 are not appreciably inflated by this mechanism. Eleven of the 19 panel metabolites were selected independently in all five folds. These are benzoic acid, C0, C10:2, Fumaric Acid, Glucose, Lysine, LysoPC a C16:0, LysoPC a C18:1, LysoPC a C18:2, Methionine, and SM C18:0. Their selection does not depend on access to the full dataset.

## 3. Discussion

The principal finding of this study is that nearly half (44.2%) of the metabolites shared between a lung and a breast cancer platform panel carry smoking-attributable variance exceeding their cancer-attributable variance. The two-way ANOVA framework assigns each metabolite a partition label that directly informs feature selection, and restricting classifiers to Signal C metabolites reduced the panel from 129 to 19 metabolites without a detectable change in never-smoker AUC.

**Figure 5:**
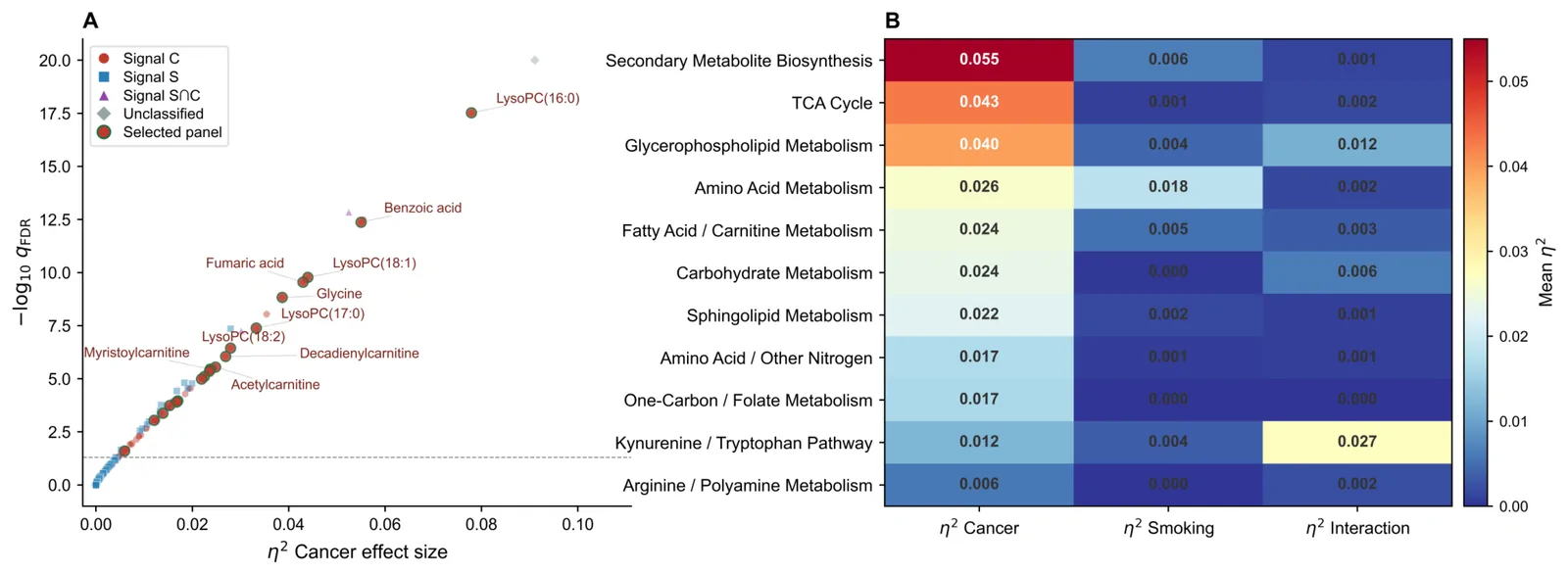
Cancer signal strength and pathway-level effects. (**A**) Volcano-style plot displaying cancer effect size ($\eta^{2}$) versus statistical significance ($-\log_{10}$ FDR). Metabolites such as LysoPC(16:0), Homovanillic Acid, Benzoic Acid, and Fumaric Acid exhibit both large effect sizes and strong statistical significance, defining the Signal C set. (**B**) Pathway-level decomposition for selected panel metabolites, showing that cancer-associated variance is concentrated in secondary metabolite biosynthesis, TCA cycle, glycerophospholipid metabolism, and amino acid metabolism. Smoking contributions remain consistently lower across pathways.

Signal C metabolites cluster in a region of low $\eta_{\mathrm{smoking}}^{2}$ and elevated $\eta_{\mathrm{cancer}}^{2}$ (Figure 2A), separated from Signal S metabolites, and only two metabolites cross both thresholds simultaneously, indicating that cancer-attributable and smoking-attributable variance are largely non-overlapping at the metabolite level in this cohort. The $\eta^{2}$ decomposition for the selected 19-metabolite panel (Figure 2B) shows a dominant cancer component with uniformly small smoking and interaction contributions. The volcano representation (Figure 5A) shows that top-ranked metabolites such as LysoPC a C16:0 and Benzoic Acid combine large cancer effect sizes with FDR well below 0.05.

### 3.1. Clinical Relevance of the Performance Difference

The improvement of 0.012 AUC points in never-smoker classification is small and is not statistically significant. Stratum-wise testing with correction for the three comparisons finds no significant difference in any stratum. The inversion combines a small non-significant gain in never-smokers with a somewhat larger non-significant loss in ever-smokers. Nonetheless, the Signal C restriction removes 85% of the metabolite measurements while leaving discrimination statistically unchanged in every stratum, with a small non-significant reduction overall (0.922 to 0.916). This behavior is reproduced at every $\eta^{2}$ threshold between 0.01 and 0.06 and survives nesting of the entire selection procedure inside the resampling loop. For a targeted assay this trade may still be worth making, since assay cost, run time, and analytical validation burden scale with panel size, and a 19-metabolite panel is more tractable than a 129-metabolite one.

The two-way ANOVA decomposition provides an interpretable partition of a shared metabolite panel into cancer-attributable and smoking-attributable components, and the classification experiments establish that a panel restricted to the cancer-attributable fraction retains never-smoker discrimination while shedding most of its measurements. Establishing clinical utility would require, at minimum, an external cohort, a prospective design, and a demonstrated accuracy advantage that this study does not provide.

### 3.2. Implications of the Combined Lung–Breast Design

Lung and breast carcinomas differ in tissue of origin, driver biology, and metabolic phenotype, and the plasma metabolome reflects those differences. A pooled model must therefore learn a decision boundary that separates cases from controls across two partly different case distributions, which is a harder problem than either single-cohort task. In practice this means the reported AUCs are likely to be conservative relative to what a tumor-type-specific model would achieve on the same data, and it means that a metabolite entering the panel is one whose case–control shift is directionally consistent in both cohorts, since metabolites with opposing shifts would cancel and lose effect size in the pooled ANOVA.

This filtering effect is visible in the composition of the selected panel, which is dominated by lipid and energy-metabolism species, Lysophosphatidylcholines, Acylcarnitines, Fumarate, and Glucose, processes altered in malignancy generally.

The cohort membership is correlated with both smoking status (82.5% ever-smokers in the lung cohort vs. 34.9% in the breast cohort) and with sex, since the breast cohort is exclusively female. A metabolite that differs between the two cohorts for reasons of tumor type, sex, or recruitment could in principle contribute apparent cancer signal. The ANOVA model partitions cancer and smoking variance within the pooled cohort, so a metabolite driven purely by cohort membership would load on whichever factor is most collinear with it, which is smoking, and would be assigned to Signal S. In addition, the never-smoker stratum in which the primary result is measured draws 47% of its members from the lung cohort and 53% from the breast cohort, so it is the most balanced stratum in the dataset. Cohort accounts for a non-trivial share of variance across the panel as a whole (median $\eta_{\mathrm{cohort}}^{2}=0.027$, maximum 0.572), and for several metabolites outside the panel it dominates, such as Taurine, C16-OH, and Spermine each carrying $\eta_{\mathrm{cohort}}^{2}$ above 0.20, and their apparent cancer effects largely disappear on adjustment (Taurine 0.052 to 0.020; C16-OH 0.028 to 0.001; Spermine 0.018 to 0.000). Agreement between the adjusted and unadjusted Signal C sets is partial (37 metabolites in common, 54 unadjusted versus 61 adjusted), so the decomposition as a whole is sensitive to cohort adjustment.

The selected panel is affected less than the full metabolite set. Seventeen of the 19 panel members remain Signal C after adjustment. The two that do not are LysoPC a C26:0 and Proline. The highest-ranked panel members carry little cohort variance and retain most of their cancer effect (LysoPC a C16:0, $\eta_{\mathrm{cohort}}^{2}=0.017$, cancer effect 0.078 to 0.055; LysoPC a C18:1, 0.014, 0.044 to 0.027). The adjusted result is a sensitivity analysis showing that tumor-type and demographic structure contribute measurably to the raw decomposition. Tumor-type-stratified evaluation shows that the panel does not perform equally in the two cohorts. In the lung cohort alone the panel achieved an overall AUC of 0.923 and a never-smoker AUC of 0.892. In the breast cohort alone the corresponding values were 0.861 and 0.864. The 0.06 difference in overall AUC indicates that the pooled estimate of 0.916 is carried disproportionately by the lung cohort, which contributes 77% of the combined sample. Notably, the never-smoker estimate is the weaker of the two in the breast cohort despite that cohort supplying the majority of never-smokers, so the pooled never-smoker result cannot be attributed principally to breast-cohort performance. Both stratified estimates rest on small case counts and should be treated as indicative.

### 3.3. Smoking Status as a Model Input

Retaining smoking status as an input may appear to be in tension with the goal of separating smoking-related from cancer-related signal. The two operations act on different objects. The decomposition removes tobacco-responsive metabolites from the feature space. The covariate supplies exposure status to the model as observed information, which removes the model’s incentive to reconstruct that status from residual tobacco-responsive variation among the retained analytes. SHAP is consistent with this reading, ranking smoking status first in overall importance (mean |ϕ|=0.605) marginally above LysoPC a C16:0 (0.601).

The cost was quantified by evaluating the identical 19-metabolite panel with the smoking feature removed. Never-smoker AUC fell from 0.910 to 0.893, overall AUC from 0.916 to 0.901, and the AUC gap reverted from −0.012 to +0.008. At 0.893 the panel cannot be distinguished from the all-metabolite model without smoking status, which also gives 0.893, or from the all-metabolite baseline with it, which gives 0.895. The reported behavior belongs to the Signal C panel and the smoking covariate together and not to the metabolite panel alone.

### 3.4. Biological Interpretation of the Selected Panel

The associations described below are cross-sectional and were not designed to test mechanism. The text distinguishes throughout between what the present analysis establishes, namely that a metabolite differs between cases and controls with a given effect size and shows negligible smoking-attributable variance, and the mechanistic accounts proposed in the prior literature to explain such differences.

#### 3.4.1. Glycerophospholipids

Five Lysophosphatidylcholine species entered the composite-rank panel: LysoPC a C16:0, C18:1, C18:2, C17:0, and C26:0. LysoPC a C16:0 was the highest-ranked Signal C metabolite across all four selection methods, with the largest $\eta_{\mathrm{cancer}}^{2}$ (0.078), negligible smoking variance ($\eta_{\mathrm{smoking}}^{2}=0.0015$), and an MI ratio of $5.2\times 10^{7}$. Its LASSO coefficient was negative, indicating lower concentrations in cases. Reduced circulating LysoPC species are among the most consistently replicated metabolomic findings in lung cancer [7,9,10] and have also been reported in breast cancer plasma across hormone receptor subtypes [11], in some cases preceding clinical diagnosis [12]. Proposed explanations invoke altered phospholipase A2 activity and increased uptake of LysoPCs as membrane precursors by proliferating tumor cells [13]. The present data are consistent with the reported direction but do not adjudicate between mechanisms. Case–control separation within never-smokers is significant for LysoPC a C16:0 (p<0.001) but not for LysoPC a C18:2 (Figure 8A,C).

#### 3.4.2. Acylcarnitines and Energy Metabolism

Free Carnitine (C0), Valerylcarnitine (C5), Myristoylcarnitine (C14), Acetylcarnitine (C2), and Decadienoylcarnitine (C10:2) were selected by ≥3 of 4 methods. Acylcarnitines mediate fatty acid transport across the inner mitochondrial membrane, and their plasma profiles are widely interpreted as reflecting the balance between fatty acid oxidation and the glycolytic shift characteristic of tumor metabolism [14,15,16,17,18]. Short- and medium-chain species have been reported as elevated in lung and gastric cancer plasma [19,20,21], and subtype-dependent acylcarnitine dysregulation has been described in breast cancer [22,23,24,25]. In the present cohort C0 was elevated in cases in the SHAP analysis, and $\eta_{\mathrm{smoking}}^{2}$ was at or below 0.010 for all five acylcarnitine panel members (C0 0.0017, C2 0.0034, C5 0.0007, C10:2 0.0062, C14 0.0100).

Fumaric Acid ranked second among metabolites by SHAP importance (mean |ϕ|=0.507). Fumarate is a TCA cycle intermediate whose plasma elevation has been attributed to impaired fumarate hydratase activity or to reverse flux through a truncated TCA cycle in hypoxic tumor regions [26,27]. Elevated plasma Fumarate has been documented in lung and breast cancer without fumarate hydratase mutations [28,29,30]. At the pathway level the TCA group carried a mean $\eta_{\mathrm{cancer}}^{2}$ of 0.043 against a mean $\eta_{\mathrm{smoking}}^{2}$ of 0.001 (Figure 5B). Glucose was selected by all four methods, consistent with reports of elevated plasma Glucose across tumor types [31] and with altered systemic Glucose handling under the Warburg effect [32].

#### 3.4.3. Amino Acids, Sphingolipids, and Microbial Metabolites

Glycine, Lysine, Methionine, Proline, and Tryptophan reflect the broad amino acid reprogramming described in proliferating tumors [33,34]. Glycine depletion is a recurrent finding across tumor types and is usually related to nucleotide and glutathione demand [35]. Methionine is discussed in terms of one-carbon and S-adenosylmethionine cycling [36], and Lysine alterations have been reported in lung adenocarcinoma plasma [37,38,39]. Tryptophan depletion is commonly attributed to diversion toward the Kynurenine pathway [40,41,42]. Smoking-attributable variance is low for all five, including Lysine (Figure 8F).

SM C18:0 was selected by all four methods with a LASSO-stable positive coefficient. Elevated plasma Sphingomyelins have been interpreted as reflecting impaired Ceramide-mediated apoptotic signaling in both breast and lung tumors [43,44,45]. Benzoic Acid, also selected by all four methods (LASSO coefficient +0.111 at C=0.05), is a product of aromatic amino acid catabolism partly mediated by gut microbial hippurate metabolism [46], and altered benzoate cycling has been reported in lung cancer [47] and among metabolites associated with cancer incidence in prospective breast cancer analyses [48].

#### 3.4.4. Pathway-Level Structure

**Figure 6:**
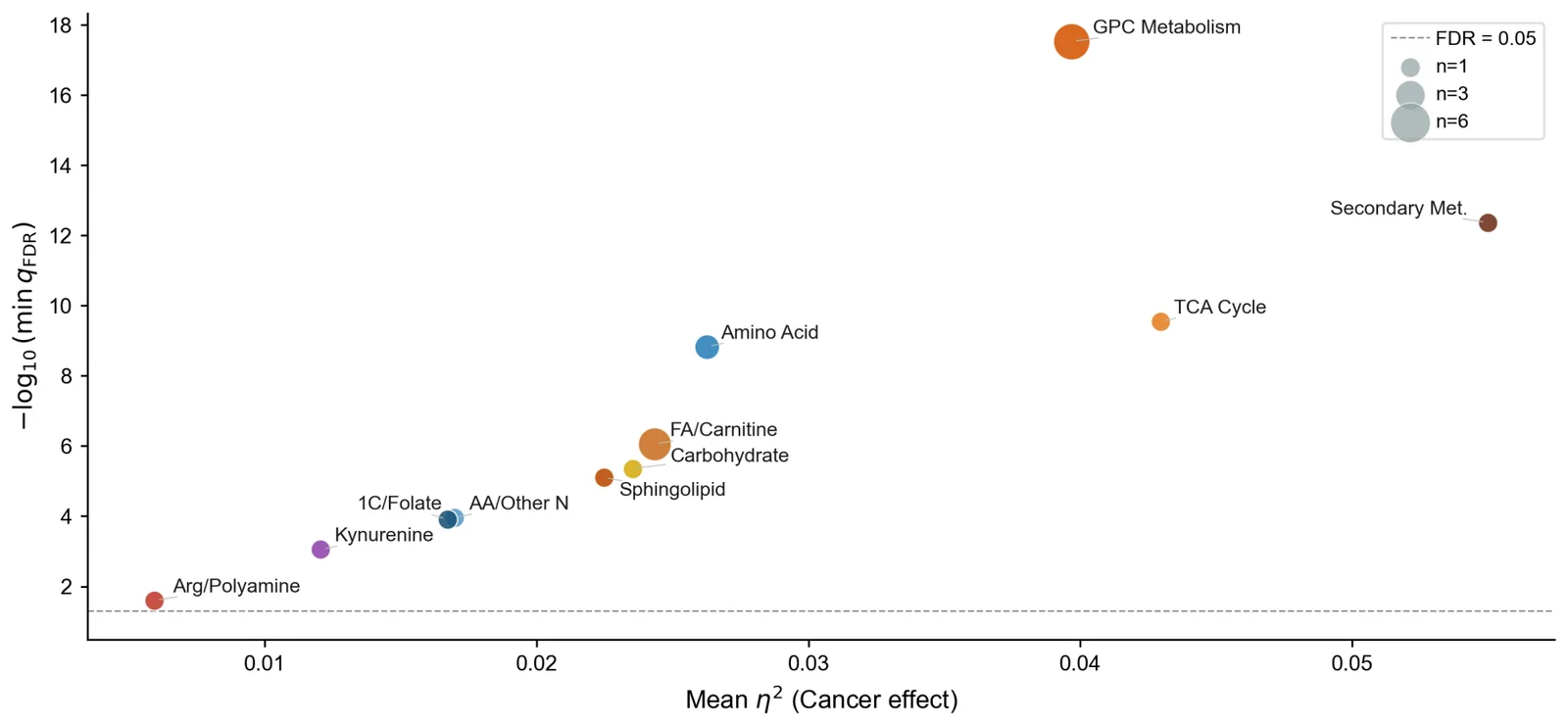
Pathway enrichment for the selected 19-metabolite panel. Bubble plot showing pathway enrichment based on mean cancer effect size ($\eta^{2}$) and statistical significance ($-\log_{10}$ FDR), with bubble size proportional to metabolite count. Glycerophospholipid metabolism (GPC), TCA Cycle, and secondary metabolite biosynthesis emerge as the most enriched pathways with the strongest cancer-associated effects.

KEGG annotation assigned the 19 panel metabolites to 11 pathway categories. Secondary metabolite biosynthesis (mean $\eta_{\mathrm{cancer}}^{2}=0.055$) and the TCA cycle (0.043) carry the largest per-pathway cancer effect sizes. Glycerophospholipid metabolism is both the most enriched and the most represented category (Figure 6), which is the clearest pathway-level signal the panel supports.

**Figure 7:**
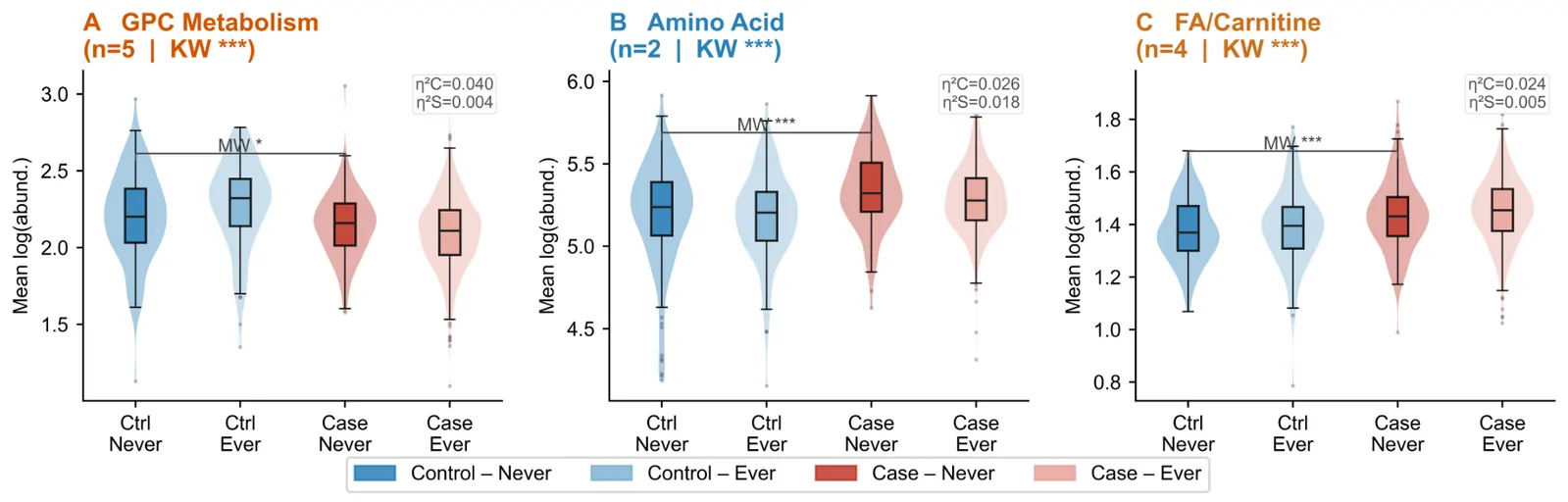
Metabolite abundance patterns within the metabolic pathways represented in the selected panel. Amino acid metabolism, glycerophospholipid metabolism (GPC), and fatty acid/Carnitine (FA/Carnitine) metabolism—case–control differences are consistently observed in never-smokers. Statistical annotations (Kruskal–Wallis and Mann–Whitney tests) indicate significant cancer-associated shifts. KW, Kruskal–Wallis test across the four cancer x smoking groups; MW, Mann–Whitney U test comparing cases and controls within the indicated stratum; * *p* < 0.05, *** *p* < 0.001.

**Figure 8:**
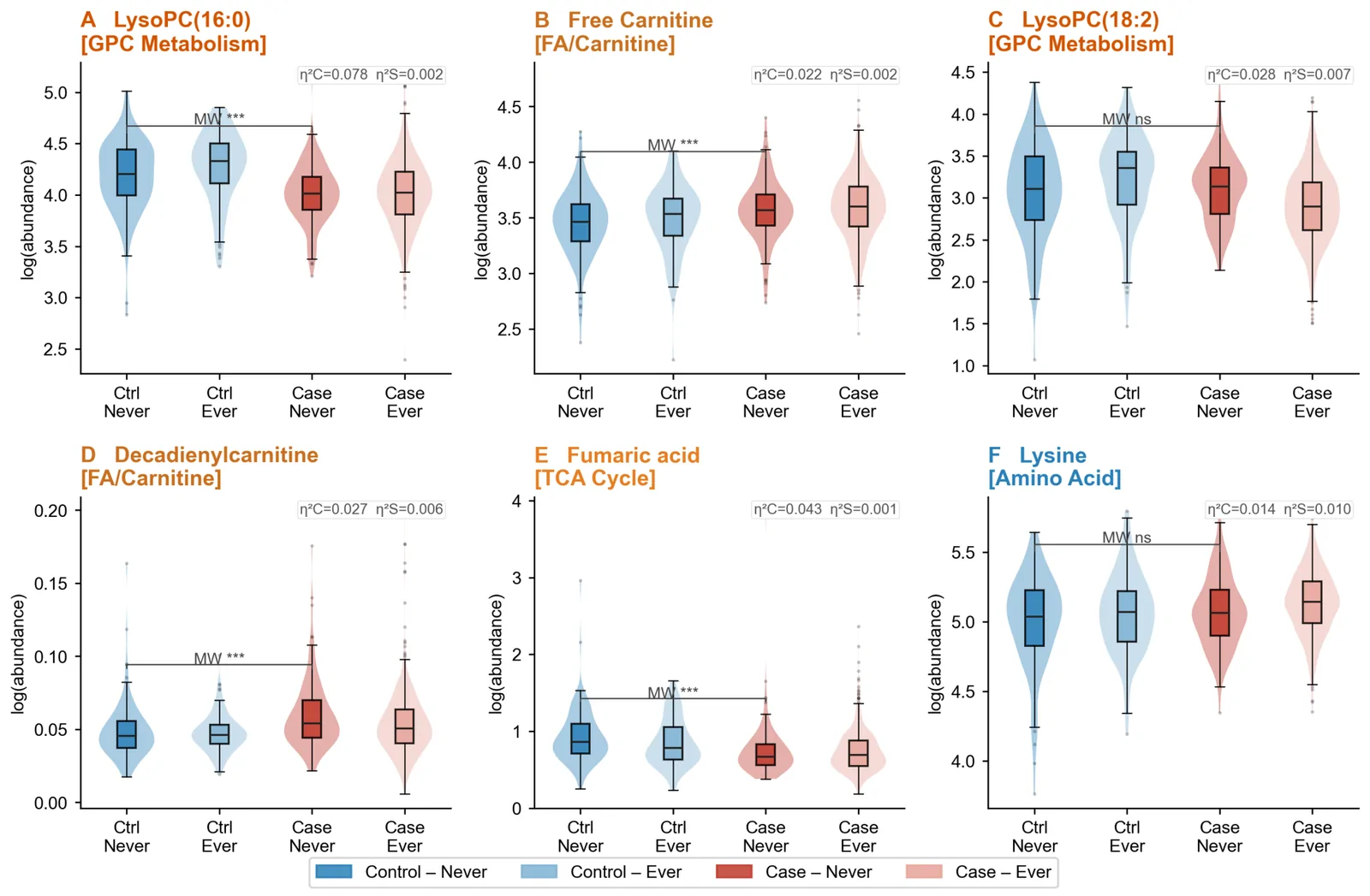
Smoking-stratified abundance of selected panel metabolites in composite rank order. Differences between cases and controls are observed within never-smokers (Mann–Whitney tests), indicating that these metabolites discriminate cancer status independently of smoking status in this cohort. Cancer effect sizes ($\eta_{C}^{2}$) are consistently larger than smoking effects ($\eta_{S}^{2}$). MW, Mann–Whitney U test comparing cases and controls within the never-smoker stratum; *** *p* < 0.001; ns, not significant.

Within-pathway smoking stratification (Figure 7) shows that case–control separation is maintained in never-smokers across pathways, with Mann–Whitney effect sizes in the never-smoker subgroup comparable to those in ever-smokers. Within the never-smoker stratum LysoPC a C16:0, Free Carnitine, Decadienoylcarnitine, and Fumaric Acid each differ significantly between cases and controls (Mann–Whitney p<0.001) (Figure 8A,B,D,E). At the pathway level the aggregate difference is significant for glycerophospholipid metabolism (p<0.05), amino acid metabolism (p<0.001), and fatty acid and Carnitine metabolism (p<0.001) (Figure 7).

The low smoking-attributable variance of the selected metabolites indicates that the panel captures cancer-associated variation that is not explained by tobacco exposure in this cohort. Whether this reflects tumor biology that is genuinely independent of smoking, as opposed to exposure effects not captured by ever/never variable, cannot be determined from these data.

## 4. Materials and Methods

### 4.1. Study Cohorts and Dataset Construction

Two independent metabolomics cohorts were combined for this analysis. The lung cancer cohort comprised 800 participants (586 cases, 214 controls); 82.5% were current or former smokers. The breast cancer cohort comprised 238 female participants (185 cases, 53 controls; 34.9% ever-smokers). Males were excluded given the breast cancer context, with the consequence that cohort membership is partially confounded with sex. Smoking status was encoded as binary (ever/never) based on self-report in both cohorts.

Both cohorts were profiled on the same Biocrates AbsoluteIDQ p180 Kit platform at the Toronto Metabolomics Innovation Centre (TMIC). Raw concentration values below the lower limit of detection (LOD) were imputed with the LOD value from the instrument calibration file. Metabolites with greater than 30% missingness were excluded prior to analysis. Remaining missing values were imputed using *k*-nearest neighbors imputation (k=2, uniform weights).

Cohort integration resulted in a dataset of 129 metabolites from n=1038 patients, 771 cancer cases (74.3%), 743 ever-smokers (71.6%), 295 never-smokers (28.4%). The full analytical pipeline is summarized in Figure 1.

### 4.2. Two-Way ANOVA Signal Decomposition

For each of the 129 shared metabolites, we fitted the two-way ANOVA model:

(1) $$\log\left(\mathrm{concentration}+1\right)∼C\left(\mathrm{cancer}\right)+C\left(\mathrm{smoking}\right)+C\left(\mathrm{cancer}\right)\times C\left(\mathrm{smoking}\right)$$

Partial eta-squared ($\eta^{2}$) was computed as ${\mathrm{SS}}_{\mathrm{effect}}/{\mathrm{SS}}_{\mathrm{total}}$ (Type II sums of squares) for each of the three terms. Raw *p*-values were corrected for multiple comparisons using the Benjamini–Hochberg false discovery rate (FDR) procedure [49].

Metabolites were assigned to one of four signal classes:

- **Signal C** (cancer-specific): $q_{\mathrm{cancer}}<0.05$ and $\eta_{\mathrm{smoking}}^{2}<0.03$;
- **Signal S** (smoking-specific): $q_{\mathrm{smoking}}<0.05$ and $\eta_{\mathrm{cancer}}^{2}<0.03$;
- **Signal S∩C** (shared): $q_{\mathrm{interaction}}<0.05$;
- **Unclassified**: none of the above criteria met.

The $\eta^{2}<0.03$ guard on the secondary axis prevents misclassification of metabolites with modest cross-effects so that a metabolite is classified as smoking-affected only when tobacco exposure accounts for appreciably more than a negligible share of its variance. Because any such convention is to some degree arbitrary, the entire pipeline was repeated across the threshold range $\eta^{2}\in \left\{0.01,0.02,0.03,0.04,0.05,0.06\right\}$ and the stability of both the Signal C membership and the downstream never-smoker AUC assessed. Note that the q<0.05 significance criterion is applied independently of this threshold, so a metabolite must satisfy both a statistical and an effect-size condition to be assigned to Signal C.

### 4.3. Feature Selection and Model Training

All four selection methods were applied exclusively to the 54 Signal C metabolites. Metabolite concentrations were log-transformed (log(x+1)) and standardized (*z*-score) prior to all downstream analyses.

RFECV was performed using a Random Forest estimator (300 trees, maximum depth = 8, minimum samples per leaf = 3). The optimal feature count was determined by five-fold stratified cross-validation maximizing AUC. The minimum feature set size was constrained to three.

$L_{1}$-penalized logistic regression was fitted at ten regularization strengths spanning C∈{0.005,0.01,0.02,0.05,0.1,0.2,0.5,1.0,2.0,5.0}. Features retaining non-zero coefficients at C=0.05 (approximately 78% of features eliminated) were designated as LASSO-stable and ranked by absolute coefficient magnitude at C=1.0 for downstream sweeping.

An XGBoost classifier (300 estimators, maximum depth = 4, learning rate = 0.05, subsampling = 0.8, column subsampling = 0.8) was trained on the full Signal C set. Features were ranked by mean absolute SHAP value.

Mutual information (MI) between each Signal C metabolite and (i) the cancer label and (ii) the smoking label was estimated using 100 permutations. Features satisfying MI(cancer)>MI(smoking) were designated as cancer-dominant and ranked by their MI ratio MI(cancer)/MI(smoking).

To reduce dependence on any single ranking method, the top 25 metabolites from each of the four ranked lists were combined by set union to form a candidate pool. Each metabolite in the pool was assigned a composite importance score equal to the mean of its normalized rank position across all four methods, where rank position was normalized by dividing by *N*, the total number of Signal C metabolites (N=54). Metabolites not appearing in a given method’s top-25 were assigned a penalty rank of N+1 before normalization. The pool was sorted in ascending order of composite score to yield a single aggregated ranked list, which was subjected to a feature-count sweep procedure to identify the optimal set of biomarkers.

Smoking status was appended to every candidate subset and was exempt from elimination by any selection method. The ANOVA decomposition removes tobacco-driven *metabolites* from the feature space, while the smoking covariate supplies exposure status to the *model* as observed information, removing the model’s incentive to reconstruct exposure from residual tobacco-responsive variation among the retained analytes.

All models were evaluated by five-fold stratified cross-validation with a fixed random seed (42) and identical fold assignments across every configuration, preserving the case–control ratio within each fold. Smoker, and never-smoker AUCs were computed separately within each held-out fold and averaged across the five folds. Reported standard errors are the standard error of the mean across folds. Subgroup AUCs were computed only where the held-out fold contained at least five subgroup members of both classes.

### 4.4. Limitations

The cross-sectional case–control design precludes causal inference, and analytical validation, calibration, and clinical operating points remain to be established in an independent external cohort. The cohort is enriched for cancer cases at 74.3% relative to any screening population, sex could not be modeled separately because the breast cohort is female only, and smoking was captured only as a self-reported binary variable, leaving residual confounding by dose, duration, and recency. Adding cohort, age, and body mass index as covariates changes Signal C membership substantially, with 37 of 54 metabolites in common.

## 5. Conclusions

Applying two-way ANOVA-based signal decomposition to a combined lung–breast cancer metabolomics dataset, we found that approximately 44% of a shared 129-metabolite panel carried smoking-attributable variance exceeding its cancer-attributable variance. This partition is the principal contribution of the study. A 19-metabolite Signal C panel with smoking status as an input feature achieved a never-smoker AUC of 0.907 against 0.895 for the all-metabolite baseline, but after correction for the three strata tested, no stratum showed a significant difference between the models. Removing the smoking covariate abolished the effect. The panel retains discrimination while requiring 85% fewer metabolite measurements. These results are internal to a single combined cohort and derive from cross-validation within the discovery data. Validation in an external cohort, ideally a prospectively collected never-smoker population with harmonized sample handling, is a prerequisite for any further claim. 

## Figures and Tables

**Table 1:** Cohort characteristics of the combined multi-cancer dataset.

Characteristic	Lung Cohort	Breast Cohort	Combined
Total patients	800	238	1038
Cancer cases	586 (73.3%)	185 (77.7%)	771 (74.3%)
Controls	214 (26.7%)	53 (22.3%)	267 (25.7%)
Ever-smokers	660 (82.5%)	83 (34.9%)	743 (71.6%)
Never-smokers	140 (17.5%)	155 (65.1%)	295 (28.4%)
Shared metabolites	—	—	129

**Table 2:** Sensitivity of the full analytical pipeline to the $\eta^{2}$ threshold separating Signal C from Signal S. The overlap column reports how many of the 19 primary panel metabolites were recovered. The all-metabolite baseline never-smoker AUC is 0.895. AUCs in this table are computed on pooled out-of-fold predictions. The corresponding fold-mean at the primary threshold is 0.910 (Table 3).

$\eta^{2}$ Threshold	Signal C (*n*)	Signal S (*n*)	Panel Overlap	Never-Smoker AUC
0.01	41	49	15/19	0.907
0.02	50	58	15/19	0.906
0.03 (primary)	54	57	19/19	0.907
0.04	59	53	14/19	0.901
0.05	61	51	16/19	0.908
0.06	63	51	16/19	0.900

## Data Availability

Dataset and predictions are not publicly available because they contain protected human-subject data governed by Institutional Review Board (IRB)-approved protocols and applicable privacy restrictions. The scripts for calculation of confidence scores are available upon request.

## References

[1] Aslam M.A., Iqbal H., Ilyas K., Rehman K., Hussain A., Akash M.S.H., Shahid M., Chen S. (2025). Metabolomic insights into smoking-induced metabolic dysfunctions: A comprehensive analysis of lipid and amino acid metabolomes. Metabolites.

[2] Kumar U., Jahnavi G., Biswas B., Alam B., Varshney S. (2026). Molecular Effect of Tobacco on Genetic, Epigenetic, and Metabolic Pathways During Cancer Progression. Cureus.

[3] Bell D.W., Brannigan B.W., Matsuo K., Finkelstein D.M., Sordella R., Settleman J., Mitsudomi T., Haber D.A. (2008). Increased prevalence of EGFR-mutant lung cancer in women and in East Asian populations: Analysis of estrogen-related polymorphisms. Clin. Cancer Res..

[4] Mitsudomi T. (2014). Molecular epidemiology of lung cancer and geographic variations with special reference to EGFR mutations. Transl. Lung Cancer Res..

[5] Jee S.H., Kim M., Kim M., Kang M., Seo Y.W., Jung K.J., Lee S.J., Hong S., Lee J.H. (2016). Clinical relevance of glycerophospholipid, sphingomyelin and glutathione metabolism in the pathogenesis of pharyngolaryngeal cancer in smokers: The Korean Cancer Prevention Study-II. Metabolomics.

[6] Cui M., Wang Q., Chen G. (2016). Serum metabolomics analysis reveals changes in signaling lipids in breast cancer patients. Biomed. Chromatogr..

[7] Klupczynska A., Plewa S., Kasprzyk M., Dyszkiewicz W., Kokot Z.J., Matysiak J. (2019). Serum lipidome screening in patients with stage I non-small cell lung cancer. Clin. Exp. Med..

[8] Kowalczyk T., Kisluk J., Pietrowska K., Godzien J., Kozlowski M., Reszeć J., Sierko E., Naumnik W., Mróz R., Moniuszko M. (2021). The ability of metabolomics to discriminate non-small-cell lung cancer subtypes depends on the stage of the disease and the type of material studied. Cancers.

[9] Zhang L., Liu X., Liu Y., Yan F., Zeng Y., Song Y., Fang H., Song D., Wang X. (2023). Lysophosphatidylcholine inhibits lung cancer cell proliferation by regulating fatty acid metabolism enzyme long-chain acyl-coenzyme A synthase 5. Clin. Transl. Med..

[10] Dong J., Cai X., Zhao L., Xue X., Zou L., Zhang X., Liang X. (2010). Lysophosphatidylcholine profiling of plasma: Discrimination of isomers and discovery of lung cancer biomarkers. Metabolomics.

[11] Qiu Y., Zhou B., Su M., Baxter S., Zheng X., Zhao X., Yen Y., Jia W. (2013). Mass spectrometry-based quantitative metabolomics revealed a distinct lipid profile in breast cancer patients. Int. J. Mol. Sci..

[12] Buentzel J., Klemp H.G., Kraetzner R., Schulz M., Dihazi G.H., Streit F., Bleckmann A., Menck K., Wlochowitz D., Binder C. (2021). Metabolomic profiling of blood-derived microvesicles in breast cancer patients. Int. J. Mol. Sci..

[13] Li L., Zhan Q., Yi K., Chen N., Li X., Yang S., Hou X., Zhao J., Yuan X., Kang C. (2022). Engineering Lipusu with lysophosphatidylcholine for improved tumor cellular uptake and anticancer efficacy. J. Mater. Chem. B.

[14] Zhang J., Wu G., Zhu H., Yang F., Yang S., Vuong A.M., Li J., Zhu D., Tao W. (2022). Circulating Carnitine Levels and Breast Cancer: A Matched Case-Control Study. Front. Oncol..

[15] Melone M.A.B., Valentino A., Margarucci S., Galderisi U., Giordano A., Peluso G. (2018). The carnitine system and cancer metabolic plasticity. Cell Death Dis..

[16] Sabbah A., Delouya G., Laskine M., Taussky D. (2025). Metabolic plasticity in prostate cancer: The Warburg effect and its clinical relevance. Am. J. Clin. Oncol..

[17] Farahzadi R., Hejazi M.S., Molavi O., Pishgahzadeh E., Montazersaheb S., Jafari S. (2023). Clinical significance of carnitine in the treatment of cancer: From traffic to the regulation. Oxidative Med. Cell. Longev..

[18] Catanese S., Beuchel C.F., Sawall T., Lordick F., Brauer R., Scholz M., Ceglarek U., Hacker U.T. (2021). Biomarkers related to fatty acid oxidative capacity are predictive for continued weight loss in cachectic cancer patients. J. Cachexia Sarcopenia Muscle.

[19] Enooku K., Nakagawa H., Fujiwara N., Kondo M., Minami T., Hoshida Y., Shibahara J., Tateishi R., Koike K. (2019). Altered serum acylcarnitine profile is associated with the status of nonalcoholic fatty liver disease (NAFLD) and NAFLD-related hepatocellular carcinoma. Sci. Rep..

[20] Li S., Gao D., Jiang Y. (2019). Function, detection and alteration of acylcarnitine metabolism in hepatocellular carcinoma. Metabolites.

[21] Ursu Ș., Ursu C.P., Bogos L.G., Pralea I.E., Moldovan R.C., Zaharie F., Spârchez Z., Ciocan R.A., Pop R.S., Bodea C.I. (2025). Exploratory Insights into Gastric Cancer Metabolism Through Amino Acid and Acylcarnitine Profiling in Plasma Samples. Biomedicines.

[22] Bauer B.A., Schmidt C.M., Ruddy K.J., Olson J.E., Meydan C., Schmidt J.C., Smith S.Y., Couch F.J., Earls J.C., Price N.D. (2024). A Multiomics, Molecular Atlas of Breast Cancer Survivors. Metabolites.

[23] Lee H.P., Oh J., Lee N., Jung Y., Yum J., Kim M., Yoo M., Park J.G., Cho J.Y. (2026). Acylcarnitines in Cancer Metabolism: Mechanistic Insights and Stratification Potential. Cancers.

[24] Yu L., Li K., Xu Z., Cui G., Zhang X. (2018). Integrated omics and gene expression analysis identifies the loss of metabolite–Metabolite correlations in small cell lung cancer. Oncotargets Ther..

[25] Akkuş T., Yaprakcı Ö., Özaslan N. (2024). Evaluation of Serum Amino Acid and Carnitine Profile in Dogs with Transmissible Venereal Tumor. Harran Univ. J. Fac. Vet. Med..

[26] Pollard P., Wortham N., Tomlinson I. (2003). The TCA cycle and tumorigenesis: The examples of fumarate hydratase and succinate dehydrogenase. Ann. Med..

[27] Li A., Wang R., Zhao Y., Zhao P., Yang J. (2024). Crosstalk between epigenetics and metabolic reprogramming in metabolic dysfunction-associated steatotic liver disease-induced hepatocellular carcinoma: A new sight. Metabolites.

[28] Lu X., Zhang A., Wang H., Xu X., Chen L., Luo L. (2025). Emerging role of the TCA cycle and its metabolites in lung disease. Front. Physiol..

[29] Valcarcel-Jimenez L., Frezza C. (2023). Fumarate hydratase (FH) and cancer: A paradigm of oncometabolism. Br. J. Cancer.

[30] Chen Y.B., Brannon A.R., Toubaji A., Dudas M.E., Won H.H., Al-Ahmadie H.A., Fine S.W., Gopalan A., Frizzell N., Voss M.H. (2014). Hereditary leiomyomatosis and renal cell carcinoma syndrome–associated renal cancer: Recognition of the syndrome by pathologic features and the utility of detecting aberrant succination by immunohistochemistry. Am. J. Surg. Pathol..

[31] Cui G., Zhang T., Ren F., Feng W.M., Yao Y., Cui J., Zhu G.L., Shi Q.L. (2015). High blood glucose levels correlate with tumor malignancy in colorectal cancer patients. Med. Sci. Monit. Int. Med. J. Exp. Clin. Res..

[32] Warburg O.H. (1926). Ueber den Stoffwechsel der Tumoren.

[33] Lieu E.L., Nguyen T., Rhyne S., Kim J. (2020). Amino acids in cancer. Exp. Mol. Med..

[34] Choi B.H., Coloff J.L. (2019). The diverse functions of non-essential amino acids in cancer. Cancers.

[35] Geeraerts S.L., Heylen E., De Keersmaecker K., Kampen K.R. (2021). The ins and outs of serine and glycine metabolism in cancer. Nat. Metab..

[36] Bernasocchi T., Mostoslavsky R. (2024). Subcellular one carbon metabolism in cancer, aging and epigenetics. Front. Epigenet. Epigenom..

[37] Zhang J., Zhang C., Jiang H., Jiang H., Yuan Y. (2021). Molecular Characterization and Clinical Relevance of Lysine Acetylation Regulators in Urological Cancers. Front. Oncol..

[38] Zhao Q., Cao Y., Hu C., Hu A., Ruan L., Bo Q., Liu Q., Chen W., Tao F., Ren M. (2014). Plasma and tissue free amino acid profiles and their concentration correlation in patients with lung cancer. Asia Pac. J. Clin. Nutr..

[39] Dols M.C., López M.D., Plaza C.R., Miranda E.P., Calle S.G., Chamorro E.V., Díaz I.A., Pino A.M., García J.A., Calderón V.G. (2006). Specific alterations in the serum amino acid profile of patients with lung cancer and head and neck cancer. Oncología.

[40] Bessede A., Peyraud F., Le Moulec S., Cousin S., Cabart M., Chomy F., Rey C., Lara O., Odin O., Nafia I. (2023). Upregulation of Indoleamine 2,3-Dioxygenase 1 in Tumor Cells and Tertiary Lymphoid Structures is a Hallmark of Inflamed Non-Small Cell Lung Cancer. Clin. Cancer Res..

[41] Onesti C.E., Boemer F., Josse C., Leduc S., Bours V., Jerusalem G. (2019). Tryptophan catabolism increases in breast cancer patients compared to healthy controls without affecting the cancer outcome or response to chemotherapy. J. Transl. Med..

[42] Mandarano M., Orecchini E., Bellezza G., Vannucci J., Ludovini V., Baglivo S., Tofanetti F.R., Chiari R., Loreti E., Puma F. (2021). Kynurenine/Tryptophan ratio as a potential blood-based biomarker in non-small cell lung cancer. Int. J. Mol. Sci..

[43] Mao C. (2025). Sphingolipid metabolism dysregulation: A cause for lung cancer development, progression, and resistance to therapies. Pulm. Circ. Crit. Med..

[44] Zheng K., Chen Z., Feng H., Chen Y., Zhang C., Yu J., Luo Y., Zhao L., Jiang X., Shi F. (2019). Sphingomyelin synthase 2 promotes an aggressive breast cancer phenotype by disrupting the homoeostasis of ceramide and sphingomyelin. Cell Death Dis..

[45] Rodríguez M., Ajona D., Seijo L.M., Sanz J., Valencia K., Corral J., Mesa-Guzmán M., Pío R., Calvo A., Lozano M.D. (2021). Molecular biomarkers in early stage lung cancer. Transl. Lung Cancer Res..

[46] Lu X., Xiong L., Zheng X., Yu Q., Xiao Y., Xie Y. (2023). Structure of gut microbiota and characteristics of fecal metabolites in patients with lung cancer. Front. Cell. Infect. Microbiol..

[47] Liu L., Yang L., Zhang H., Li H., Shang T., Liu L. (2025). Lung cancer and the Gut-microbiota-lung Axis: Emerging evidence and potential clinical implications. Front. Med..

[48] Huang X., Lu S., Li X., Wu J., Zu Q., Duan Z., Luo M., Jia Y. (2025). Multi-omics profiling reveals the role of 4-ethylbenzoic acid in promoting proliferation and invasion of cervical cancer. Front. Med..

[49] Benjamini Y., Hochberg Y. (1995). Controlling the false discovery rate: A practical and powerful approach to multiple testing. J. R. Stat. Soc. Ser. B (Methodol.).

